# Therapeutic alternative of the ketogenic Mediterranean diet to improve mitochondrial activity in Amyotrophic Lateral Sclerosis (ALS): A Comprehensive Review

**DOI:** 10.1002/fsn3.1324

**Published:** 2019-12-16

**Authors:** Jordi Caplliure‐Llopis, Thalía Peralta‐Chamba, Sandra Carrera‐Juliá, María Cuerda‐Ballester, Eraci Drehmer‐Rieger, María Mar López‐Rodriguez, Jose Enrique de la Rubia Ortí

**Affiliations:** ^1^ Doctoral Degree's School Catholic University of Valencia Valencia Spain; ^2^ University Hospital la Ribera Alzira Spain; ^3^ Department of Nursing Catholic University of Valencia Valencia Spain; ^4^ Faculty of Medicine and Odontology Catholic. University of Valencia Valencia Spain; ^5^ Department of Health and Functional Valorization Catholic University of Valencia Valencia Spain; ^6^ Department of Nursing University of Almería Almería Spain

**Keywords:** amyotrophic lateral sclerosis, ketone bodies, mediterranean diet, mitochondria

## Abstract

Amyotrophic lateral sclerosis (ALS) is an incurable neurodegenerative disease which is pathogenically based on the mitochondrial alteration of motor neurons, causing progressive neuron death. While ALS is characterized by enormous oxidative stress, the Mediterranean diet has been seen to have high antioxidant power. Therefore, the aim of this study is to determine how the Mediterranean diet can improve mitochondrial activity, establishing the specific nutrients and, in addition, observing the pathogenic mechanisms related to the disease that would achieve this improvement. To this end, a comprehensive review of the literature was performed using PubMed. KBs have been observed to have a neuroprotective effect to improve energy balance, increasing survival and the number of motor neurons. This ketogenesis can be achieved after following a Mediterranean diet which is associated with great benefits in other neurodegenerative diseases such as Alzheimer's disease, Parkinson's disease, and ALS. These benefits are due to the high antioxidant power especially based on polyphenols contained mainly in olive oil, wine, nuts, or berries. In short, KBs could be considered as a promising option to treat ALS, representing an alternative source to glucose in motor neurons by providing neuroprotection. In addition, treatment results can be improved as ketogenesis can be achieved (increase in KBs) by following a Mediterranean diet, thanks to the high antioxidant properties which, at the same time, would improve the high oxidative stress that characterizes the disease.

## Graphical abstract

Amyotrophic lateral sclerosis (ALS) is an incurable neurodegenerative disease whose etiology is based on the progressive death of motor neurons. To slow the course of the pathology, the mitochondrial activity of these neurons and the high oxidative stress shown by patients should be improved. This comprehensive review aims to perform an analysis of the literature published in the PubMed database to determine whether ketone bodies, contributed by a ketogenic Mediterranean diet, could improve this pathogenesis. The conclusions drawn indicate that the Mediterranean ketogenic diet provides neuroprotection at the mitochondrial level, in turn presenting a high antioxidant capacity. This seems to indicate that this diet represents an interesting therapeutic alternative for the disease, improving the prognosis of the pathology.

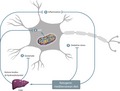

## 1 INTRODUCTION

Neurons are cells in charge of transmitting information through chemical and electrical signals, therefore requiring a high level of energy in the brain. This use of energy represents between 20% and 25% of the total oxygen consumed by the body, of which approximately half is used to generate membrane potentials. At the same time, they maintain ion gradients for neurotransmission, the main source of said percentage (Wong et al., 1995). In this sense, in the event of needing extra energy derived from, for example, an increase in synaptic activity, there is no type of energy reserve that can be used in the brain (Karbowski, 2007).

The essential energy source for neurons, in the form of adenosine triphosphate (ATP), is obtained, on the one hand, from oxygen and, on the other hand, from glucose through catabolism performed in the Krebs cycle. Glucose is used by brain gray matter ten times more than the rest of the body, while there are variations in that consumption in different regions of the brain. This could explain that, in the case of an energetic alteration in the neurons, there is a great susceptibility to neurodegeneration and a great diversity of neurodegenerative diseases that can be triggered depending on the region where this altered glucose consumption takes place (Barros et al., 2013). In any case and whatever the disease may be, the basic explanation for this is glucose hypometabolism in the brain. It produces a structural and functional imbalance of the affected part of the brain, based on mitochondrial dysfunction which ends up influencing it overall. It can aggravate other existing alterations that are the real cause of disease such as Alzheimer's, Parkinson's (Paoli, Bianco, Damiani, & Bosco, 2014), or epilepsy (Jóźwiak, Kossoff, & Kotulska‐Jóźwiak, 2011).

Regarding this pathological imbalance, the proposed therapeutic alternative is to provide an energy source that is not based on glucose, such as that represented by ketone bodies (KBs). A ketogenic diet is effective in patients with intractable epilepsy, myoclonic‐astatic epilepsy (Doose syndrome) and also in Dravet syndrome (at an early stage) or phosphofructokinase deficiency and type V glycogenosis (McArdle's disease). Moreover, a ketogenic diet may be also effective in neurodegenerative diseases, such as Alzheimer's, Parkinson's, and amyotrophic lateral sclerosis (ALS), due to its neuroprotective action, which improves the mitochondrial function by rescuing the production of ATP (Barañano & Hartman, 2008).

## 2 AMYOTROPHIC LATERAL SCLEROSIS (ALS)

Amyotrophic lateral sclerosis (ALS) is the most common motor neuron disease with an incidence of 1 in every 2,000 people (Gordon, 2013). Most cases are sporadic (sALS), and only 10% are familial (fALS) (Miller, Mitchell, Lyon, & Moore, 2003). This neurodegenerative disorder is characterized by the loss of motor neurons, both upper and lower, in the brain and spinal cord, which leads to paralysis of the voluntary muscles (Robberecht & Philips, 2013). This leads to a progressive motor dysfunction that results in alterations in the respiratory system (Polkey, Lyall, Moxham, & Leigh, 1999) until the death of the patient in a period of 2 to 5 years (Valko & Ciesla, 2019). Nowadays, there is no medical cure for this disease.

**Figure 1:**
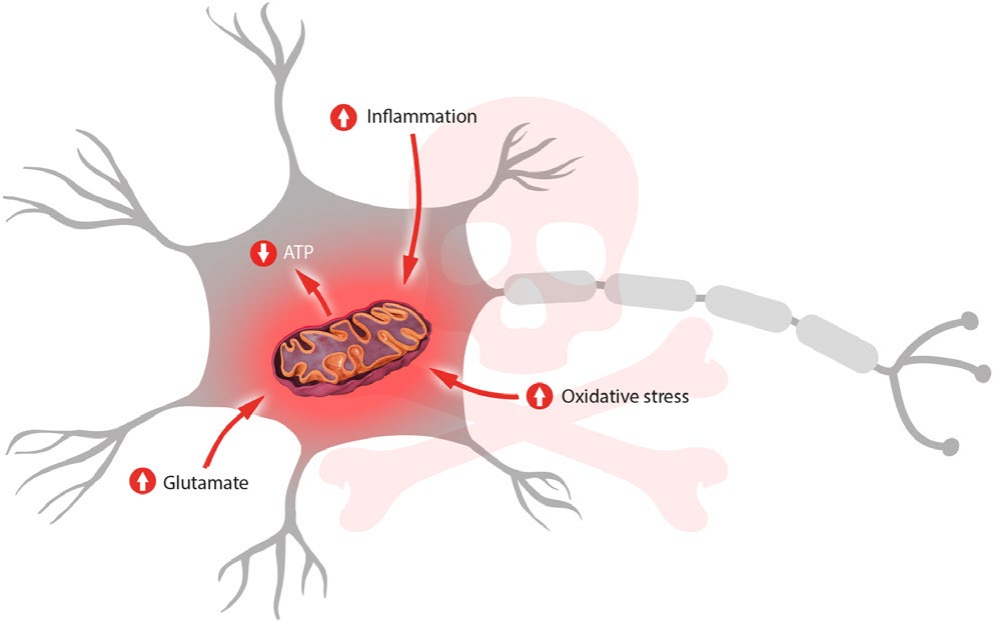
Diagram of the pathogenesis of amyotrophic lateral sclerosis (ALS) observed mainly after the genetic mutation in the SOD1 gene, and which causes the death of the motor neuron by damaging mitochondrial activity

The pathogenic mechanisms of ALS that are currently accepted (Figure 1) are loss of oxidative control with an excessive generation of oxidative free radicals, accumulation of neurofilaments, and excitotoxicity linked to an increase in the neurotransmitter glutamate, producing a mitochondrial membrane dysfunction. This dysfunction will eventually lead to an alteration in the energy balance related to a lower activity of the enzymes of the mitochondrial electron transport chain (ETC) in the spinal cord.

This is especially evident in the mutant forms of SOD1, which represent 20% of the cases of fALS. In these cases, a loss of mitochondrial membrane potential and mitochondrial inflammation are induced, with a decrease in the production of ATP, alteration of calcium homeostasis, and loss of the activity of the mitochondrial transport chain enzyme, which obviously leads to aforementioned bioenergetic alteration (Carrì et al., 1997; Ferri et al., 2006; Kruman, Pedersen, Springer, & Mattson, 1999).

Excessive oxidative stress is related to neuron death, not only as a primary factor but as a consequence of other mechanisms (Beal, Lang, & Ludolph, 2005), especially using reactive oxygen species (ROS), including superoxide, hydrogen peroxide (H_2_O_2_), hydroxyl (OH) free radicals, and nitric oxide (NO).

This high level of oxidation has been directly and especially involved in the pathophysiology of fALS through SOD1 mutation. However, it is also linked to patients with fALS, where elevated levels of 8‐hydroxy‐2‐deoxyguanosine (8‐OHdG) have been evidenced, which is a marker of oxidation of the motor cortex in patients with ALS (Ferrante et al., 2002).

The high level of oxidative stress of the two types of ALS can be explained by the high reactivity of ROS, especially with lipids, proteins, and DNA to induce cellular damage (Esposito et al., 2002; Halliwell, 2001; Singh, Sharad, & Kapur, 2004). In this sense, the large amount of lipids in the nervous tissue is worth mentioning (Singh et al., 2004), to which a high metabolic activity at a neuronal level should be also added, leading to a high ROS formation in these cells (Gurney et al., 1996).

On another note, glutamate is the primary excitatory neurotransmitter of the CNS that is necessary for neuronal synapse. However, an excess of this neurotransmitter on the synaptic cleft level due to failures in its uptake produces a prolonged opening of glutamate‐dependent Ca^++^ channels in the neuron, generating a large number of free radicals that end up damaging the mitochondrial structure (Doble, 1999; Shaw & Ince, 1997).

In addition, there is also a clear relation between mitochondrial dysfunction and motor neuron diseases. Morphological changes can be observed in mitochondria of SOD1 mice (Menzies, Ince, & Shaw, 2002) and in patients with sALS without mutations in SOD1 (Murata, Ohtsuka, & Terayama, 2008). At the same time, alterations in the ETC and mitochondrial DNA mutations in patients with ALS, related to the pathogenesis of the disease (Menzies et al., 2002), are also evidenced. Mitochondrial damage is related to an increase in intracellular Ca^++^ (Cassarino, Cassarino, Bennett, & Bennett, 1999) and alterations in the ETC (complexes I‐IV) or coenzymes (Fosslien, 2001), eventually leading to an increase in ROS production and the activation of a caspase cascade that produces greater oxidative damage (Menzies et al., 2002).

All this suggests that an improvement of mitochondrial function may represent a therapeutic approach for ALS (Ari et al., 2014). In this sense, given the great results achieved when following a Mediterranean diet in other neurodegenerative diseases (that will be broadly addressed in this paper), following said diet could be an option to improve this function. Therefore, the aim of this study is to establish how the Mediterranean diet can improve mitochondrial activity, establishing the specific nutrients and, in addition, observing by means of which pathogenic mechanisms related to the disease would achieve this improvement.

Regarding altered energy balance, the first alternative to assess could be to increase or force the hydrocarbon pathway represented by glucose or fructose. However, it seems that this alternative is not the best one due to the existence of a metabolic alteration of cerebral glucose in patients with ALS (Pradat & Dib, 2009). This has been confirmed by analyzing 1H‐NMR‐based metabolomic profiling of CSF, mainly with the purpose of certain metabolites in CSF, including KBs such as acetate or acetone (Blasco et al., 2010). In this sense, evidence confirms this alteration. It has been observed that patients with ALS are at risk of developing diabetes mellitus. This may be because of defects in the SOD1 gene, which are related to alterations in glucose metabolism, as seen in bacteria and yeast (Hamasaki et al., 2015), which lead to patients with diabetes to be more likely to develop the disease (Sun, Lu, Chen, Hou, & Li, 2015). At the same time, an early inhibition of the glycolytic capacity of muscle fibers in SOD1‐G86R mice has been recently demonstrated, observing a progressive inhibition of phosphofructokinase 1 and an induction of the expression of pyruvate dehydrogenase kinase 4 (thereby strongly inactivating pyruvate dehydrogenase) (Palamiuc et al., 2015). In short, it can be said that these patients do not energetically relate well with calories from carbohydrates, which explains the fact that they only use 84% of the calories they need on a daily basis (Heffernan et al., 2004; Kasarskis, Berryman, Vanderleest, Schneider, & McClain, 1996). This results in an increased risk of developing the disease (Okamoto et al., 2007a) and of weight loss associated with the progression of the disease (Desport et al., 2000; Paganoni, Deng, Jaffa, Cudkowicz, & Wills, 2011). As a consequence, it is necessary to propose new sources of energy and, in this sense, protein contribution does not seem to achieve an improvement in the survival and progression of the disease (Stanich, Chiapetta, Oliveria, & Gabbai, 2002). However, the fatty acid beta‐oxidation pathway decreases the glucose needs that neurons have and also generates a higher yield in ATP. Therefore, the contribution of these acids in a diet is an alternative to the glycolytic pathway. In fact, it has been proven in mice that diets based on lipid calories decrease the risk of developing the disease (Morozova et al., 2008; Okamoto et al., 2007b) and the progression of it by 90% (Mattson, Cutler, & Camandola, 2007), especially when the origin of the fat is butter in 21% (Dupuis, Oudart, Rene, de Aguilar, & Loeffler, 2004), which is the main source of KBs, together with coconut oil.

## 3 KETONE BODIES (KBs)

KBs are the molecules: acetoacetate, beta‐hydroxybutyrate, and acetone. These molecules are involved in several metabolic pathways such as beta oxidation of fatty acids, gluconeogenesis, Krebs cycle, de novo lipogenesis, and sterol biosynthesis (Cahill, 2006; McGarry & Foster, 2003). They are mainly produced in the liver, originating in fatty acid beta oxidation, and mainly from acetyl‐CoA. If there is an excess in acetyl‐CoA, it can transform into acetoacetate and then finally beta‐hydroxybutyric or directly transform into acetone (Newman & Verdin, 2014). They are then transferred to other tissues, such as brain, muscle, and heart, where the final oxidation is produced, creating energy and, therefore, representing a fuel source that is especially important for the brain (Achanta & Rae, 2017)**.**

Ketone body production can occur in physiological situations such as pregnancy or in newborns (Puchalska & Crawford, 2017). Nonetheless, KBs are mainly produced as a result of fasting or low carbohydrate diets which, in turn, involve a lower source of energy, the main one being KBs (McGarry & Foster, 2003; Robinson & Williamson, 2017).

In this sense, due to the energy alternative that KBs represent on a mitochondrial level in neurons, KBs have important neuroprotective effects (Edmond, Robbins, Bergstrom, Cole, & de Vellis, 1987; Yang, He, & Schulz, 1987). This neuroprotection activity has been demonstrated in several neurological disorders, particularly in epilepsy (Stafstrom & Rho, 2012; Vining et al., 1998), in rodent models of Parkinson's disease (PD) (Cheng et al., 2009), in pain and inflammation (Ruskin, Kawamura, & Masino, 2009), and in juvenile traumatic brain injury (Deng‐Bryant, Prins, Hovda, & Harris, 2011; Hu et al., 2009). It should be specified at this point that the contribution of KBs should not be the same for all diseases. For example, the administration of KBs required for epilepsy is high (reducing glucose metabolism and inhibiting the release of glutamate in neurons), while in Alzheimer's disease (AD) low doses of KB shave a better therapeutic effect. In AD, this fact can be explained, on the one hand, because small doses are sufficient for energy supplementation (by providing a fraction of ATP that helps compensate the alteration of glucose metabolism). On the other hand, these low doses (specifically beta‐hydroxybutyrate) have an impact on the improvement of cognitive function due to both metabolic supplementation and the inhibition of glutamate release induced by Aβ as a gliotransmitter, which most likely reduces hyperexcitability and inflammation (Hertz, Chen, & Waagepetersen, 2015). In addition, we should highlight that beta‐hydroxybutyrate is particularly used as an intermediary between the energy metabolism, but it also regulates the cell functions, partly by directly activating the G protein‐coupled receptor HCA1/GPR81, HCA2/GPR109A y HCA3/GPR109B (Ahmed, Tunaru, & Offermanns, 2009). These HCA receptors help to maintain homeostasis in changing metabolic and dietary conditions, by controlling metabolic, immune, and other bodily functions (Offermanns, 2017). The anti‐inflammatory effects must be added to this neuroprotective capacity, and in this sense, recent studies show that the activation of the HCA2 receptor (which until now was known only to produce lipolysis) by beta‐hydroxybutyrate mediates deep anti‐inflammatory effects in a variety of tissues (Lukasova, Malaval, Gille, Kero, & Offermanns, 2011; Wanders, Graff, White, & Judd, 2013). This anti‐inflammatory effect could be related to the inhibition of the release of proinflammatory cytokines, IL‐1β and IL‐18 observed thanks to beta‐hydroxybutyrate (Goldberg et al., 2017; Youm et al., 2015). This indicates that HCA2 could be an important objective to treat inflammatory processes of diseases with based on a great level of inflammation (Graff, Fang, Wanders, & Judd, 2016).

In addition, they are also related to decreasing oxidative stress. High oxidative stress is associated with mitochondrial dysfunction and with the majority of neurodegenerative diseases (Islam, 2017). Yet, in this sense, KBs induce a reduction in free radicals (Veech, 2014) in relation to those produced when the main source of energy is glucose. Therefore, this involves a neuroprotection on a mitochondrial level (Greco, Glenn, Hovda, & Prins, 2016).

Finally, they could also be related to the metabolism of glutamate that is the main excitatory neurotransmitter in the CNS. KBs are able to decrease the levels of glutamate, to accelerate its transformation into GABA that, on the other hand, represents the main inhibitory neurotransmitter and appears as a neuroprotective agent (Yudkoff, Daikhin, Nissim, Lazarow, & Nissim, 2004).

## 4 KETONE BODIES (KBs) AND AMYOTROPHIC LATERAL SCLEROSIS (ALS)

As for ALS, KBs have been used in animal models giving promising results (Table 1). In the first study published in 2006, it was observed that motor neurons were preserved by administering a ketogenic diet to transgenic mice (SOD1‐G93A) through the administration of caprylic triglycerides (contributed by fractionated coconut oil), which was associated with an increased motor performance maintenance regarding control mice following an isocaloric diet. It was also observed that weight and synthesis of ATP at a mitochondrial level were increased (Zhao et al., 2006). Subsequently, this has been confirmed in another publication in which ketogenesis was produced in transgenic animal models, showing an improvement in motor functions associated with an increase in motor neurons (Ari et al., 2014; Tefera et al., 2016; Zhao et al., 2012). Specifically, an increase in mitochondrial oxygen consumption both basal and maximum (which was correlated with an increase in KBs in the blood) and an increase in survival (they lived 6 days longer than the control mice) was observed (Zhao et al., 2012). Also, this survival increase was observed, associated with a delay in cognitive deterioration, after following the Deanna protocol based on the ketogenic diet (Ari et al., 2014). Finally, the administration of triheptanoin (triglyceride composed of 3 medium‐chain fatty acids) protected lumbar motor neurons, which allowed a delay in the appearance of motor symptoms characteristic of the disease (Tefera et al., 2016). In order to explain these specific improvements of KBs in model animals of ALS, several authors propose specific activities related to the properties detailed in the previous section for these metabolites (Table 1).

**Table 1:** Main improvements obtained on models of transgenic mice G93A‐SOD1 of amyotrophic lateral sclerosis (ALS), related in turn with the activities proposed for the KBs in other articles

Author, year	Intervention	Improvement	Proposed activity
Zhao et al., (2006)	Ketogenic diet	Preservation of motor neurons. Increase in weight and synthesis of ATP at the mitochondrial level.	Increase the antioxidant power of these endogenous antioxidants (Kong et al., 2017; Veech et al., 2017). Ability to restore the activity of complex I of the electron chain (Tieu et al., 2003; Zhao et al., 2006).
Zhao et al. (2012)	Caprylic triglyceride	Increase in motor neurons. Increase in mitochondrial oxygen consumption.	Regulation of sirtuin‐mediated responses (Körner et al., 2013; Song et al., 2013).
Ari et al. (2014)	Deanna Protocol	Delay in cognitive deterioration. Improved motor function.	Reducing hyperexcitability and inflammation (Mamelak, 2017; Steele et al., 2007).
Tefera et al. (2016)	Triglyceride Triheptanoin	Preservation of motor neurons. Survival increase.	Higher performance of Krebs cycle (Niessen et al., 2007).

On the one hand, there is glutathione peroxidase, catalase, and superoxide dismutase as endogenous antioxidants that counteract ROS damage to stop high oxidation. In this sense, KBs increase the antioxidant power of these endogenous antioxidants (Kong et al., 2017; Veech et al., 2017).

Regarding the excitability of glutamate evidenced in this disease, as already indicated, KBs decrease its levels. Thus, in adequate doses, the levels of glutamate in the synaptic cleft can be regulated, reducing hyperexcitability and inflammation, which would improve the course of the disease (Deng‐Bryant et al., 2011) as observed in AD, where cognitive improvements are achieved in both animal (Mamelak, 2017) and human (Steele, Stuchbury, & Münch, 2007) models.

Regarding energy alterations as a consequence of mitochondrial malfunction in patients with ALS, KBs increase the correct mitochondrial activity and energy production problems, possibly due to their ability to restore the activity of complex I of the electron chain (whose function is reduced in ALS) as seen for this disease, both in in vitro studies with cell cultures (Tieu et al., 2003) and in animal models (Zhao et al., 2006). In this sense, we must outline the Krebs cycle which, alongside the electron transport chain, are the necessary sources of ATP in order for cells to function and survive, and this is altered in ALS (Niessen et al., 2007). On the other hand, sirtuins (SIRTs) have been linked to different studies about the disease, since sirtuins appear altered in SOD1 G93A‐mouse models and patient tissues (Körner et al., 2013). In vitro SIRT3 protects against mitochondrial fragmentation and neuronal cell death induced by SOD1‐G93A (Song, Song, Kincaid, Bossy, & Bossy‐Wetzel, 2013) and, in this sense, primary motor neurons increase the expression of SIRTs after treatment with medium‐chain triglycerides (MCTs), the main source of KBs. Therefore, KBs can regulate mitochondrial activity and cell survival through the responses mediated by sirtuins (Körner et al., 2013). SIRT3 also regulates the production of KBs, which is confirmed by the increase in the expression of sirtuins in primary motor neuron cultures after treatment with MCTs, which can regulate mitochondrial activity and cell survival through the responses mediated by SIRTs.

In addition, making further emphasis on the enormous oxidative stress that characterizes ALS (Kruman et al., 1999), the contribution of KBs (after liver metabolism) should originate from a diet with high antioxidant power.

## 5 MEDITERRANEAN DIET

The most common version of the Mediterranean diet dates back to the 1990s and is based on plant foods, fresh fruit, olive oil, dairy products such as cheese or yogurt, fish, and poultry eaten in low to moderate amounts. It also includes the consumption of eggs (up to 4 weekly), small amounts of red meat and red wine in low to moderate amounts. The total fat in this diet represents 25%–35% of the daily intake of calories, of which 8% or less are saturated fats (Willett et al., 1995). Therefore, the Mediterranean diet is a highly varied diet mainly based on fish, olive oil, red wine, and vegetables (Alvarez‐Sala Walther, Millán Núñez‐Cortés, & de Oya Otero, 1996).

The Mediterranean diet could be a good source of KBs. In fact, the nutrients of a typical ketogenic diet are mainly nonstarchy vegetables, butter, eggs, olive oil, avocados, walnuts, and seeds (Taylor, Swerdlow, Burns, & Sullivan, 2019), many of which, such as olive oil, eggs, vegetables, and walnuts, are characteristic to the Mediterranean diet. These products, especially fish rich in omega‐3 polyunsaturated fatty acids, provide high amounts of KBs (Paoli et al., 2015). Moreover, this diet can further increase the production of KBs without being a typical ketogenic diet; enriching it with foods which contain large amounts of medium‐chain triglycerides (MCTs) such as coconut oil (Pehowich, Gomes, & Barnes, 2000), goat's milk (Haenlein, 2004), or butter characteristic of the Mediterranean diet (Dupuis et al., 2004; Lahoz et al., 2018); and proportionally decreasing the percentage of carbohydrates (Paoli, Cenci, & Grimaldi, 2011). In this sense, this combination has been proposed as the “Spanish Ketogenic Mediterranean Diet,” in which <30 g of carbohydrates per day is provided and, therefore, it fits under the definition of a ketogenic diet better (Pérez‐Guisado, Mũoz‐Serrano, & Alonso‐Moraga, 2008). This would be in line with the Keto‐Mediet approach that combines the benefits of a ketogenic and Mediterranean diet (Perng, Chen, Perng, & Jambazian, 2017).

In addition, as well as being a promising vehicle for KBs, we must highlight the great antioxidant capacity that characterizes this diet. Oxidative stress is a direct consequence of the imbalance between the production of free radicals and the antioxidant capacity of our body and has been related to the development of various diseases, especially cardiovascular, carcinogenic, and neurodegenerative diseases. Among all the existing diets that can be followed, the Mediterranean diet is possibly the richest in antioxidants. Regarding its antioxidant properties, the Mediterranean diet contains, on the one hand, soluble or low molecular weight antioxidants such as vitamins C and E, phenolic compounds and carotenoids, and other macromolecular antioxidants that are polymeric phenolic compounds or polyphenols and carotenoids linked to macromolecules of plant foods, which contribute 61% to the antioxidant capacity of the diet (Hernández‐Ruiz et al., 2018; Pérez‐Jiménez, Díaz‐Rubio, & Saura‐Calixto, 2015). All these antioxidants, associated with the practice of physical activity, increase the total antioxidant capacity (Koloverou et al., 2016).

As for the specific nutrients in the diet that provide this antioxidant capacity, the activity attributed to virgin olive oil, one of its most outstanding foods, should be highlighted. The Mediterranean diet, when enriched with virgin olive oil, improves the atheroprotective functions of HDL in humans. This is due to the fact that the antioxidants in this food can help maintain Lecithin‐cholesterol acyltransferase (LCAT), the enzyme responsible for cholesterol esterification, not oxidized and functional. In addition, there is also an increase in four key functions: cholesterol flow capacity, HDL‐C metabolism, vasoprotective effects, and anti‐inflammatory/antioxidant properties by a significant reduction in oxidative stress, which is not observed in low‐fat diets (Hernáez et al., 2017). Moreover, olive oil contains hydroxytyrosol (HT), a polyphenol that shows anticancer, anti‐inflammatory, neuroprotective, and especially antioxidant properties (Hu, He, Jiang, & Xu, 2014; de Pablos, Espinosa‐Oliva, Hornedo‐Ortega, Cano, & Arguelles, 2019). At a hepatic level, the administration of tyrosol supplements in mice produces the attenuation of hepatic lipid peroxidation and the restoration of the redox balance of the antioxidant glutathione (Kalaiselvan, Samuthirapandi, Govindaraju, Sheeja Malar, & Kasi, 2016), and it also inhibits the oxidative stress induced by palmitic acid in hepatocytes (Sarna et al., 2016). This evidence may be related to the activity of the total polyphenolic fraction (TPF) and to HT itself, which achieve a powerful in vitro free radical scavenging (improving the redox state by increasing glutathione levels (Kouka et al., 2017). It may be also related to the protection against oxidative stress at a cellular level, achieved thanks to HT. This fact has been observed in Caenorhabditis elegans nematodes, when extra virgin olive oil is included in the Mediterranean diet (Rossi et al., 2017).

Another important component of the Mediterranean diet is wine, to which important antioxidant properties are attributed in the same way. Wine contains a wide variety of phenolic compounds such as quercetin, myricetin, catechins, tannins, anthocyanidins, resveratrol, and ferulic acid (Caruana, Cauchi, & Vassallo, 2016), which have been linked to preventing or delaying the progression of intestinal diseases characterized by oxidative stress and inflammation. It acts as a scavenger of free radicals and modulators of specific genes related to inflammation and involved in cellular redox signaling (Biasi et al., 2014; Colombo et al., 2019). In addition, neuroprotective effects have also been observed in neurodegenerative diseases, not only thanks to the antioxidant property, but also through a combined ability to antagonize amyloid aggregation, suppress neuroinflammation, modulate signaling pathways, and decrease mitochondrial dysfunction (Caruana et al., 2016).

Finally, grapes, nuts, and berries, common in Mediterranean diets, contain another polyphenol such as resveratrol that activates sirtuins, which could explain some of the beneficial effects of the diet (Rodríguez‐Morató et al., 2016; Russo et al., 2014; Silva et al., 2019).

## 6 THE ANTIOXIDANT EFFECT OF THE MEDITERRANEAN DIET IN NEURODEGENERATIVE DISEASES

This enormous antioxidant capacity has led to very good results in several diseases characterized by high levels of oxidative stress. Human beings who follow a Mediterranean diet have better parameters of anthropometric, metabolic, and inflammatory risk (Dinu, Pagliai, Casini, & Sofi, 2018). In this sense, neurodegenerative diseases, among which the aforementioned AD, PE, multiple sclerosis (MS), or ALS stand out and whose etiologies are partly unknown, have a clear metabolic and inflammatory components. Therefore, the possible early interventions that can be carried out in these diseases are mainly related to lifestyle habits, and here, diet plays a key role. In relation to the Mediterranean diet, a high adherence to this diet is associated with a low mortality and a lower risk of developing chronic diseases such as cancer, metabolic syndrome, depression, cardiovascular, and neurodegenerative diseases (Anderson & Nieman, 2016; Chedraui & Pérez‐López, 2013; Estruch, 2014) such as AD, where the risk is reduced by almost 10%, as oxidative stress, inflammation, and beta‐amyloid accumulation decrease (Samadi, Moradi, Moradinazar, Mostafai, & Pasdar, 2019). Among individuals who present mild cognitive impairment, a greater adherence to this diet reduces the risk of progression to AD by 48% (Dussaillant, Echeverría, Urquiaga, Velasco, & Rigotti, 2016), by covering their energy requirements (Henderson et al., 2009). Furthermore, the Mediterranean diet is associated with a lower probability of PE, and a low adherence to it with an earlier age of the onset of the disease (Alcalay et al., 2012).

Additionally, all these benefits are mainly based on its antioxidant activity. Evidence of oxidative stress in neurodegenerative diseases indicates the potential role of antioxidants in the Mediterranean diet (Russo et al., 2014). In this sense, the phenols of *Oleaeuropaea L*., found in extra virgin olive oil and that exert strong antioxidant properties, are able to counteract oxidative stress in the brain tissue and reduce inflammation producing a certain protective effect in AD, PE, MS, and ALS (Rodríguez‐Morató et al., 2016). In fact, oleuropein and HT (as discussed above) act as direct eliminators of free radicals; HT and oleocanthal are strong inhibitors of cyclooxygenases (COX), and oleuropein counteracts the oxidation of low‐density lipoproteins (LDL) (Angeloni, Malaguti, Barbalace, & Hrelia, 2017), which would explain the benefit of consumption while suffering from these diseases.

This means that oxidative stress must be taken into account, not as an isolated process, but associated with other related aspects or deriving in part from it, specifically highlighting the inflammatory process. A risk factor for chronic diseases including neurodegenerative diseases is low‐grade inflammation, characterized by high concentrations of inflammatory markers in the absence of overt symptoms. The intake of polyphenols reduces low‐grade inflammation, as opposed to Western or meat‐based diets, which on the contrary are associated with adverse health outcomes possibly due to the high content of proinflammatory foods and nutrients (Bonaccio et al., 2017; Medina‐Remón, Kirwan, Lamuela‐Raventós, & Estruch, 2018).

As for the activity of polyphenols, it has been observed that those found in virgin olive oil interfere in different ways in the amyloidosis that is fundamentally based on incorrect folding and aggregation of a series of peptides at an intra‐ and extracellular level, affecting cell physiology and viability. Specifically, polyphenols reduce this aggregation and its cytotoxic effects (Rigacci & Stefani, 2016).

Moreover, there are also findings in animal and human models that polyphenols may have a role in regulating neurotrophin levels, in particular nerve growth factor (NGF) and brain‐derived neurotrophic factor (BDNF), suggesting that polyphenols can induce their protective effects by potentiating the action of neurotrophins that stimulate growth, proliferation, survival, and neuronal differentiation (Carito et al., 2016).

In addition, among polyphenols, anthocyanins belong to the family of bioactive compounds called flavonoids (responsible for the red, purple, and blue colors of wine and many fruits, vegetables, and cereals). These are beneficial against a series of ischemic and degenerative conditions, being able to observe how anthocyanin supplementation counteracts the inflammatory response to stress conditions (Cerletti et al., 2017; Hornedo‐Ortega et al., 2018). At the same time, polyphenols, together with B vitamins (folic acid, vitamin B6, and vitamin B12), which are also found in large quantities in this diet, decrease homocysteine concentrations that are elevated in patients with Alzheimer's disease (Zoccolella et al., 2008). Furthermore, oleuropein aglycone (OLE), enriched in extra virgin olive oil, protects against neurodegeneration in AD. In a study on OLE treatment, this decreased neurotoxicity and Aβ‐induced cognitive impairment by reducing plaque burden and consistency, as a result of a strong induction of autophagy with a recovery of the lysosomal system (whose dysfunction is one of the first alterations that occur in the disease) and the activation of microglia (Grossi et al., 2013). These findings confirm the benefits of the Mediterranean diet in AD, not only in slowing down its evolution, but also in preventing the disease. Dietary supplements such as polyphenols, B vitamins and polyunsaturated fatty acids are beneficial to improve the pathogenesis and the development of AD. Moreover, consuming fish, fruits, vegetables, coffee, and alcohol in moderation reduces the risk of the disease onset. All these components are a fundamental part of the Mediterranean diet and highlight its ability to reduce the accumulation of beta‐peptide amyloid (Aβ) and oxidative stress (Hu et al., 2014).

But, we do not only find polyphenol‐type antioxidants in the Mediterranean diet. This diet also stands out for providing antioxidants of another nature such as tocopherols especially contained in nuts, lettuce, peas, wheat germ, vegetable oils, eggs, grapes, and wine, which act by deactivating the reactive species in their initial stages, preventing the oxidative process from continuing (Aguiló et al., 2005). Or also carotenoids found in strongly pigmented fruits and vegetables, such as carrots, tomatoes, and red peppers, react with peroxyl radicals to form another less reactive radical that may react with another peroxyl radical and finally lead to a nonreactive compound (Tur, 2004).

On the other hand, several beverages contain various natural compounds called phytochemicals, which exert antitumor, antiangiogenic, and antioxidant properties. Their consumption is related to better cognitive functions. Phytochemicals are found in different beverages: epigallocatechin in green tea, triterpenoids in citrus juices, xanthohumol in beer, procyanidin in chocolate, and caffeine and resveratrol in red wine. All these drinks are fundamental elements in the Mediterranean diet (Rossi et al., 2014). In particular, resveratrol is shown as one of the most promising antioxidants contained in the Mediterranean diet for ALS disease. This is due to the fact that improvements have been obtained at the cognitive level and increased survival of motor neurons by generating a great expression and activation of the SIRT1 and AMPK (Mancuso et al., 2014). This adds to the properties already demonstrated at the inflammatory level, by inhibiting the expression of certain cytokines and tumor necrosis factor (TNF). Resveratrol is also an antioxidant through the indirect activation of the Nrf2 pathway (regulatory protein of genes that produce antioxidant enzymes capable of neutralizing reactive oxygen species) (Alcalay et al., 2012; Angeloni et al., 2017).

Finally, the main fatty acids of the Mediterranean diet (oleic acid and docosahexaenoic acid) seem to attenuate the main toxic effects of 7‐ketocholesterol (7KC) that occur due to the auto‐oxidation of cholesterol. 7KC is found in plasma and/or cerebrospinal fluid of patients with neurodegenerative diseases and is believed to contribute to the activation of microglial cells involved in neurodegeneration (Debbabi et al., 2017; Johnson et al., 2008).

After analyzing the main works published in recent years with intervention in humans, animal models, or in vitro (Table 2), it can be concluded that the Mediterranean diet presents great benefits to treat and prevent oxidative stress in most neurodegenerative diseases.

**Table 2:** Antioxidant effects of the Mediterranean diet in neurodegenerative diseases associated with nutrients

Author, year	Nutrient; antioxidant provided	Study design	Study Population	Proposed antioxidant activity
**Virgin olive oil**				
*Polyphenols (Hidroxitirosol, oleuropein aglycone)*				
Kalaiselvan et al. (2016)	Olive oil; phenolic compounds (hydroxyl‐tyrosol and tyrosol).	Randomized study.	Male Wistar rats.	Attenuation of hepatic lipid peroxidation and restoration of the redox balance of the antioxidant glutathione. Hydroxytyrosol and Tyrosol produce GSH activation in liver homogenates.
Kouka et al. (2017)	Olive oil (greek Olea europea variety); total polyphenolic fraction and hydroxyl‐tyrosol.	In vitro test.	Endothelial cells and murine C2C12 myoblasts.	Free radical scavenging. Improved redox status by increasing glutathione levels.
Grossi et al. (2013)	Extra virgin olive oil; Oleuropein aglycone.	Randomized study.	TgCRND8 mice.	Decreased neurotoxicity by induction of autophagy and recovery of the lysosomal system. OLE reduces astrocyte reaction, reducing inflammation.
**Fish and dairy products**				
*B Vitamins (B12 and B9)*				
Zoccolella et al. (2008)	Folate (vitamin B9) y vitamin B12.	Transversal study.	People suffering from ALS.	Decreased homocysteine concentration causing inflammatory damage and oxidation when in excess. Improvement of atrophy and mitochondrial activity in myocytes.
**Citrus juices, beer, red wine, green tea**				
*Phytochemicals (epigallocatechin, triterpenoids, xanthohumol, procyanidin, resveratrol)*				
Mancuso et al. (2014)	Resveratrol.	Randomized clinical study.	SOD1 (G93A) mice.	Promotes the survival of motor neurons by increasing Sirt1 activity. Greater expression and activation of SIRT 1 and AMPK. Resveratrol treatment normalizes autophagic flux. Resveratrol suppresses the activation of the NF‐kB pathway in LPS‐activated microglia by reducing the phosphorylation and consequent degradation of its inhibitor (IkB).
**Oily fish, eggs, seafood, dairy, nuts, vegetables and fruits**				
*Docosahexaenoic acid*				
Debbabi et al. (2017)	Fatty fish (sardines); docosahexaenoic acid (DHA).	Transversal study.	BV‐2 murine microglial cells.	Attenuation of the toxic effects of 7‐ketocholesterol (7KC from self‐oxidation of cholesterol) using unsaturated fatty acids, based on the ability of an exogenous supply of unsaturated fatty acids to reduce the degradation of endogenous fatty acids.

**Figure 2:**
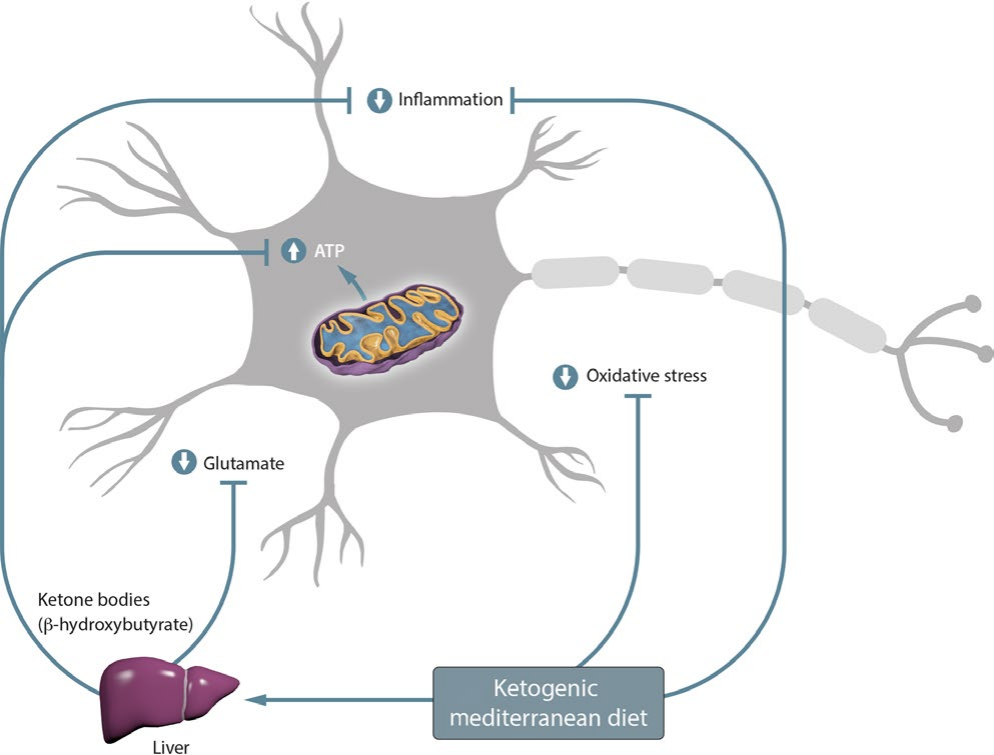
Activity proposal of the Mediterranean ketogenic diet on the different pathogenic mechanisms of amyotrophic lateral sclerosis (ALS)

All these antioxidant properties linked to its inflammatory activity and, alongside their capacity to produce KBs after hepatic beta oxidation as explained in this paper, make them a promising therapeutic strategy for ALS, by blocking the main pathogenic mechanisms of the disease that damage mitochondrial activity, being able to slow down the death of the motor neuron (Figure 2).

## 7 CONCLUSIONS

The seriousness of ALS and the absence of a curative treatment make both the improvement in the evolution of the pathology, as well as in the prevention of its appearance, a challenge of current medicine. Consequently, taking into account the mitochondrial dysfunction in these patients and the main cause of motor neuron degeneration, it seems that a ketogenic alternative is promising since it interferes with the main pathogenic mechanisms of the disease.

In addition, it is interesting how ketogenesis can be produced, not only by following the typical ketogenic diet, but also alongside Mediterranean diet with adaptations such as food rich in MCTs, as in coconut oil, or a decrease in carbohydrates. This means there is a high source of KBs represented in a diet that, in addition, is characterized by great antioxidant properties, allowing to reduce the enormous level of inflammation derived mainly from oxidative stress evidenced at a neuronal level in ALS. In this sense, the main nutrients and antioxidants of the diet are analyzed in this study, outlining the activity of polyphenols contained mainly in olive oil and red wine (main bastions of the diet), which through different mechanisms manage to diminish the oxidation and inflammation of these pathologies.

## 8 KEY FINDINGS

Ketone bodies, provided through a Mediterranean diet enriched with triglyceride‐rich foods of medium chain and low carbohydrate, could be a therapeutic alternative for improving the pathogenesis of amyotrophic lateral sclerosis (ALS). These improvements are due to the neuroprotective capacity of these metabolites and the enormous antioxidant power of the Mediterranean diet due mainly to the polyphenols provided by nutrients such as olive oil, wine, nuts, or berries.

## CONFLICT OF INTEREST

The author declares that I do not have any conflict of interest.

## ETHICAL APPROVAL

This study does not involve any human or animal testing.

## References

[fsn31324-bib-0001] Achanta, L. B. , & Rae, C. D. (2017). β‐Hydroxybutyrate in the brain: One molecule. Multiple Mechanisms. Neurochemical Research, 42(1), 35–49. 10.1007/s11064-016-2099-2 27826689

[fsn31324-bib-0002] Aguiló, A. , Tauler, P. , Fuentespina, E. , Tur, J. A. , Córdova, A. , & Pons, A. (2005). Antioxidant response to oxidative stress induced by exhaustive exercise. Physiology & Behavior, 84(1), 1–7. 10.1016/j.physbeh.2004.07.034 15642600

[fsn31324-bib-0003] Ahmed, K. , Tunaru, S. , & Offermanns, S. (2009). GPR109A, GPR109B and GPR81, a family of hydroxy‐carboxylic acid receptors. Trends in Pharmacological Sciences, 30(11), 557–562. 10.1016/j.tips.2009.09.001 19837462

[fsn31324-bib-0004] Alcalay, R. N. , Gu, Y. , Mejia‐Santana, H. , Cote, L. , Marder, K. S. , & Scarmeas, N. (2012). The association between Mediterranean diet adherence and Parkinson's disease. Movement Disorders, 27(6), 771–774. 10.1002/mds.24918 22314772 PMC3349773

[fsn31324-bib-0005] Alvarez‐Sala Walther, L. A. , Millán Núñez‐Cortés, J. , & de Oya Otero, M. (1996). The Mediterranean diet in Spain. Legend or reality? (II). Other elements in the Mediterranean diet: Vegetables and fruits, fish. Evolution of the diet and cardiovascular diseases in Spain in the last decades. Revista Clinica Espanola, 196(9), 636–646. 8966325

[fsn31324-bib-0006] Anderson, J. J. B. , & Nieman, D. C. (2016). Diet quality—the greeks had it right!. Nutrients, 8(10), 636 10.3390/nu8100636 PMC5084023 27754409

[fsn31324-bib-0007] Angeloni, C. , Malaguti, M. , Barbalace, M. C. , & Hrelia, S. (2017). Bioactivity of olive oil phenols in neuroprotection. International Journal of Molecular Sciences, 18, 10.3390/ijms18112230 PMC5713200 29068387

[fsn31324-bib-0008] Ari, C. , Poff, A. M. , Held, H. E. , Landon, C. S. , Goldhagen, C. R. , Mavromates, N. , & D'Agostino, D. P. (2014). Metabolic therapy with deanna protocol supplementation delays disease progression and extends survival in amyotrophic lateral sclerosis (ALS) mouse model. PLoS ONE, 9(7), e103526 10.1371/journal.pone.0103526 25061944 PMC4111621

[fsn31324-bib-0009] Barañano, K. W. , & Hartman, A. L. (2008). The ketogenic diet: Uses in epilepsy and other neurologic illnesses. Current Treatment Options in Neurology, 10(6), 410–419. 10.1007/s11940-008-0043-8. 18990309 PMC2898565

[fsn31324-bib-0010] Barros, L. F. , San Martín, A. , Sotelo‐Hitschfeld, T. , Lerchundi, R. , Fernández‐Moncada, I. , Ruminot, I. , … Espinoza, D. (2013). Small is fast: Astrocytic glucose and lactate metabolism at cellular resolution. Frontiers in Cellular Neuroscience, 7, 27 10.3389/fncel.2013.00027 23526722 PMC3605549

[fsn31324-bib-0011] Beal, M. F. , Lang, A. E. , & Ludolph, A. C. (2005). Neurodegenerative diseases: Neurobiology, pathogenesis and therapeutics (Vol. 77). Cambridge, UK: Cambridge University Press.

[fsn31324-bib-0012] Biasi, F. , Deiana, M. , Guina, T. , Gamba, P. , Leonarduzzi, G. , & Poli, G. (2014). Wine consumption and intestinal redox homeostasis. Redox Biology, 2, 795–802. 10.1016/j.redox.2014.06.008 25009781 PMC4085343

[fsn31324-bib-0013] Blasco, H. , Corcia, P. , Moreau, C. , Veau, S. , Fournier, C. , Vourc'h, P. , … Andres, C. R. (2010). 1H‐NMR‐Based metabolomic profiling of CSF in early amyotrophic lateral sclerosis. PLoS ONE, 5(10), e13223 10.1371/journal.pone.0013223 20949041 PMC2951909

[fsn31324-bib-0014] Bonaccio, M. , Pounis, G. , Cerletti, C. , Donati, M. B. , Iacoviello, L. , & de Gaetano, G. (2017). Mediterranean diet, dietary polyphenols and low grade inflammation: Results from the MOLI‐SANI study. British Journal of Clinical Pharmacology, 83, 107–113. 10.1111/bcp.12924 26935858 PMC5338145

[fsn31324-bib-0015] Cahill, G. F. J. (2006). Fuel metabolism in starvation. [Review] [82 refs]. Annual Review of Nutrition, 26, 1–22. 10.1146/annurev.nutr.26.061505.111258 16848698

[fsn31324-bib-0016] Carito, V. , Ceccanti, M. , Tarani, L. , Ferraguti, G. , Chaldakov, G. N. , & Fiore, M. (2016). Neurotrophins' modulation by olive polyphenols. Current Medicinal Chemistry, 23(28), 3189–3197. 10.2174/0929867323666160627104022 27356540

[fsn31324-bib-0017] Carrì, M. T. , Ferri, A. , Battistoni, A. , Famhy, L. , Gabbianelli, R. , Poccia, F. , & Rotilio, G. (1997). Expression of a Cu, Zn superoxide dismutase typical of familial amyotrophic lateral sclerosis induces mitochondrial alteration and increase of cytosolic Ca2+ concentration in transfected neuroblastoma SH‐SY5Y cells. FEBS Letters, 414(2), 365–368. 10.1016/S0014-5793(97)01051-X 9315720

[fsn31324-bib-0018] Caruana, M. , Cauchi, R. , & Vassallo, N. (2016). Putative role of red wine polyphenols against brain pathology in Alzheimer's and Parkinson's disease. Frontiers in Nutrition, 3, 31 10.3389/fnut.2016.00031 27570766 PMC4981604

[fsn31324-bib-0019] Cassarino, D. S. , Cassarino, D. S. , Bennett, J. P. , & Bennett, J. P. (1999). An evaluation of the role of mitochondria in neurodegenerative diseases: Mitochondrial mutations and oxidative pathology, protective nuclear responses, and cell death in neurodegeneration. Brain Research Reviews, 29(1), 1–25. 9974149 10.1016/s0165-0173(98)00046-0

[fsn31324-bib-0020] Cerletti, C. , De Curtis, A. , Bracone, F. , Digesù, C. , Morganti, A. G. , Iacoviello, L. , … Donati, M. B. (2017). Dietary anthocyanins and health: Data from FLORA and ATHENA EU projects. British Journal of Clinical Pharmacology, 83(1), 103–106. 10.1111/bcp.12943 27016122 PMC5338134

[fsn31324-bib-0021] Chedraui, P. , & Pérez‐López, F. R. (2013). Nutrition and health during mid‐life: Searching for solutions and meeting challenges for the aging population. Climacteric, 16(S1), 85–95. 10.3109/13697137.2013.802884 23651240

[fsn31324-bib-0022] Cheng, B. , Yang, X. , An, L. , Gao, B. , Liu, X. , & Liu, S. (2009). Ketogenic diet protects dopaminergic neurons against 6‐OHDA neurotoxicity via up‐regulating glutathione in a rat model of Parkinson's disease. Brain Research, 1286, 25–31. 10.1016/j.brainres.2009.06.060 19559687

[fsn31324-bib-0023] Colombo, F. , Di Lorenzo, C. , Regazzoni, L. , Fumagalli, M. , Sangiovanni, E. , Peres De Sousa, L. , … Dell'Agli, M. (2019). Phenolic profiles and anti‐inflammatory activities of sixteen table grape (: Vitis vinifera L.) varieties. Food and Function, 10(4), 1797–1807. 10.1039/c8fo02175a 30778463

[fsn31324-bib-0024] de Pablos, R. M. , Espinosa‐Oliva, A. M. , Hornedo‐Ortega, R. , Cano, M. , & Arguelles, S. (2019). Hydroxytyrosol protects from aging process via AMPK and autophagy; a review of its effects on cancer, metabolic syndrome, osteoporosis, immune‐mediated and neurodegenerative diseases. Pharmacological Research, 143, 58–72. 10.1016/j.phrs.2019.03.005 30853597

[fsn31324-bib-0025] Debbabi, M. , Zarrouk, A. , Bezine, M. , Meddeb, W. , Nury, T. , Badreddine, A. , … Lizard, G. (2017). Comparison of the effects of major fatty acids present in the Mediterranean diet (oleic acid, docosahexaenoic acid) and in hydrogenated oils (elaidic acid) on 7‐ketocholesterol‐induced oxiapoptophagy in microglial BV‐2 cells. Chemistry and Physics of Lipids, 207(Pt B), 151–170. 10.1016/j.chemphyslip.2017.04.002 28408132

[fsn31324-bib-0026] Deng‐Bryant, Y. , Prins, M. L. , Hovda, D. A. , & Harris, N. G. (2011). Ketogenic diet prevents alterations in brain metabolism in young but not adult rats after traumatic brain injury. Journal of Neurotrauma, 28(9), 1813–1825. 10.1089/neu.2011.1822 21635175 PMC3172875

[fsn31324-bib-0027] Desport, J. C. , Preux, P. M. , Truong, C. T. , Courat, L. , Vallat, J. M. , & Couratier, P. (2000). Nutritional assessment and survival in ALS patients. Amyotrophic Lateral Sclerosis and Other Motor Neuron Disorders: Official Publication of the World Federation of Neurology, Research Group on Motor Neuron Diseases, 1(2), 91–96. 10.1080/14660820050515386 11467055

[fsn31324-bib-0028] Dinu, M. , Pagliai, G. , Casini, A. , & Sofi, F. (2018). Mediterranean diet and multiple health outcomes: An umbrella review of meta‐analyses of observational studies and randomised trials. European Journal of Clinical Nutrition, 72, 30–43. 10.1038/ejcn.2017.58 28488692

[fsn31324-bib-0029] Doble, A. (1999). The role of excitotoxicity in neurodegenerative disease: Implications for therapy. Pharmacology & Therapeutics, 81(3), 163–221. 10334661 10.1016/s0163-7258(98)00042-4

[fsn31324-bib-0030] Dupuis, L. , Oudart, H. , Rene, F. , de Aguilar, J.‐L.‐G. , & Loeffler, J.‐P. (2004). Evidence for defective energy homeostasis in amyotrophic lateral sclerosis: Benefit of a high‐energy diet in a transgenic mouse model. Proceedings of the National Academy of Sciences of the United States of America, 101(30), 11159–11164. 10.1073/pnas.0402026101 15263088 PMC503756

[fsn31324-bib-0031] Dussaillant, C. , Echeverría, G. , Urquiaga, I. , Velasco, N. , & Rigotti, A. (2016). Evidencia actual sobre los beneficios de la dieta mediterránea en salud. Revista Médica De Chile, 144(8), 990–997. 10.4067/s0034-98872016000800012 27905651

[fsn31324-bib-0032] Edmond, J. , Robbins, R. A. , Bergstrom, J. D. , Cole, R. A. , & de Vellis, J. (1987). Capacity for substrate utilization in oxidative metabolism by neurons, astrocytes, and oligodendrocytes from developing brain in primary culture. Journal of Neuroscience Research, 18(4), 551–561. 10.1002/jnr.490180407 3481403

[fsn31324-bib-0033] Esposito, E. , Rotilio, D. , Di Matteo, V. , Di Giulio, C. , Cacchio, M. , & Algeri, S. (2002). A review of specific dietary antioxidants and the effects on biochemical mechanisms related to neurodegenerative processes. Neurobiology of Aging, 23(5), 719–735. 10.1016/S0197-4580(02)00078-7 12392777

[fsn31324-bib-0034] Estruch, R. (2014). Cardiovascular mortality: how can it be prevented? Nefrologia, 34(5), 561–569. 10.3265/Nefrologia.pre2014.Apr.12481 25036262

[fsn31324-bib-0035] Ferrante, R. J. , Browne, S. E. , Shinobu, L. A. , Bowling, A. C. , Baik, M. J. , MacGarvey, U. , … Beal, M. F. (2002). Evidence of increased oxidative damage in both sporadic and familial amyotrophic lateral sclerosis. Journal of Neurochemistry, 69(5), 2064–2074. 10.1046/j.1471-4159.1997.69052064.x 9349552

[fsn31324-bib-0036] Ferri, A. , Cozzolino, M. , Crosio, C. , Nencini, M. , Casciati, A. , Gralla, E. B. , … Carri, M. T. (2006). Familial ALS‐superoxide dismutases associate with mitochondria and shift their redox potentials. Proceedings of the National Academy of Sciences of the United States of America, 103(37), 13860–13865. 10.1073/pnas.0605814103 16945901 PMC1557633

[fsn31324-bib-0037] Fosslien, E. (2001). Mitochondrial medicine–molecular pathology of defective oxidative phosphorylation. Annals of Clinical and Laboratory Science, 31(1), 25–67. 11314862

[fsn31324-bib-0038] Goldberg, E. L. , Asher, J. L. , Molony, R. D. , Shaw, A. C. , Zeiss, C. J. , Wang, C. , … Dixit, V. D. (2017). β‐Hydroxybutyrate deactivates neutrophil NLRP3 inflammasome to relieve gout flares. Cell Reports, 18(9), 2077–2087. 10.1016/j.celrep.2017.02.004 28249154 PMC5527297

[fsn31324-bib-0039] Gordon, P. (2013). Amyotrophic lateral sclerosis: An update for 2013 clinical features, pathophysiology, management and therapeutic trials. Aging and Disease, 04(05), 295–310. 10.14336/AD.2013.0400295 PMC3794725 24124634

[fsn31324-bib-0040] Graff, E. C. , Fang, H. , Wanders, D. , & Judd, R. L. (2016). Anti‐inflammatory effects of the hydroxycarboxylic acid receptor 2. Metabolism: Clinical and Experimental, 65, 102–113. 10.1016/j.metabol.2015.10.001 26773933

[fsn31324-bib-0041] Greco, T. , Glenn, T. C. , Hovda, D. A. , & Prins, M. L. (2016). Ketogenic diet decreases oxidative stress and improves mitochondrial respiratory complex activity. Journal of Cerebral Blood Flow and Metabolism, 36(9), 1603–1613. 10.1177/0271678X15610584 26661201 PMC5012517

[fsn31324-bib-0042] Grossi, C. , Rigacci, S. , Ambrosini, S. , Ed Dami, T. , Luccarini, I. , Traini, C. , Stefani, M. (2013). The polyphenol oleuropein aglycone protects TgCRND8 mice against Aß plaque pathology. PLoS ONE, 8(8), e71702 10.1371/journal.pone.0071702 23951225 PMC3738517

[fsn31324-bib-0043] Gurney, M. E. , Cutting, F. B. , Zhai, P. , Doble, A. , Taylor, C. P. , Andrus, P. K. , & Hall, E. D. (1996). Benefit of vitamin E, riluzole, and gabapentin in a transgenic model of familial amyotrophic lateral sclerosis. Annals of Neurology, 39(2), 147–157. 10.1002/ana.410390203 8967745

[fsn31324-bib-0044] Haenlein, G. F. W. (2004). Goat milk in human nutrition. Small Ruminant Research, 51(2), 155–163. 10.1016/j.smallrumres.2003.08.010

[fsn31324-bib-0045] Halliwell, B. (2001). Role of free radicals in the neurodegenerative diseases: Therapeutic implications for antioxidant treatment. Drugs and Aging, 18, 685–716. 10.2165/00002512-200118090-00004 11599635

[fsn31324-bib-0046] Hamasaki, H. , Takeuchi, Y. , Masui, Y. , Ohta, Y. , Abe, K. , Yoshino, H. , & Yanai, H. (2015). Development of diabetes in a familial amyotrophic lateral sclerosis patient carrying the I113T SOD1 mutation. Neuroendocrinology Letters, 36(5), 414–416. 26707039

[fsn31324-bib-0047] Heffernan, C. , Jenkinson, C. , Holmes, T. , Feder, G. , Kupfer, R. , Leigh, P. N. , … Sidhu, P. (2004). Nutritional management in MND/ALS patients: An evidence based review. Amyotrophic Lateral Sclerosis and Other Motor Neuron Disorders, 5, 72–83. 10.1080/14660820410020349 15204009

[fsn31324-bib-0048] Henderson, S. T. , Vogel, J. L. , Barr, L. J. , Garvin, F. , Jones, J. J. , & Costantini, L. C. (2009). Study of the ketogenic agent AC‐1202 in mild to moderate Alzheimer's disease: A randomized, double‐blind, placebo‐controlled, multicenter trial. Nutrition and Metabolism, 6(1), 31 10.1186/1743-7075-6-31 19664276 PMC2731764

[fsn31324-bib-0049] Hernáez, Á. , Castañer, O. , Elosua, R. , Pintó, X. , Estruch, R. , Salas‐Salvadó, J. , … Fitó, M. (2017). Mediterranean diet improves high‐density lipoprotein function in high‐cardiovascular‐risk individuals. Circulation, 135(7), 633–643. 10.1161/CIRCULATIONAHA.116.023712 28193797

[fsn31324-bib-0050] Hernández‐Ruiz, A. , García‐Villanova, B. , Guerra‐Hernández, E. , Amiano, P. , Sánchez, M. J. , Dorronsoro, M. , & Molina‐Montes, E. (2018). Comparison of the Dietary Antioxidant Profiles of 21 a priori Defined Mediterranean Diet Indexes. Journal of the Academy of Nutrition and Dietetics, 118(12), 2254–2268.e8. 10.1016/j.jand.2018.01.006 29580874

[fsn31324-bib-0051] Hertz, L. , Chen, Y. , & Waagepetersen, H. S. (2015). July). Effects of ketone bodies in Alzheimer's disease in relation to neural hypometabolism, β‐amyloid toxicity, and astrocyte function. Journal of Neurochemistry, 134, 7–20. 10.1111/jnc.13107 25832906

[fsn31324-bib-0052] Hornedo‐Ortega, R. , Cerezo, A. B. , de Pablos, R. M. , Krisa, S. , Richard, T. , García‐Parrilla, M. C. , & Troncoso, A. M. (2018). Phenolic compounds characteristic of the mediterranean diet in mitigating microglia‐mediated neuroinflammation. Frontiers in Cellular Neuroscience, 12, 373 10.3389/fncel.2018.00373 30405355 PMC6206263

[fsn31324-bib-0053] Hu, T. , He, X. W. , Jiang, J. G. , & Xu, X. L. (2014). Hydroxytyrosol and its potential therapeutic effects. Journal of Agricultural and Food Chemistry, 62, 1449–1455. 10.1021/jf405820v 24479643

[fsn31324-bib-0054] Hu, Z. G. , Wang, H. D. , Qiao, L. , Yan, W. , Tan, Q. F. , & Yin, H. X. (2009). The protective effect of the ketogenic diet on traumatic brain injury‐induced cell death in juvenile rats. Brain Injury, 23(5), 459–465. 10.1080/02699050902788469 19408168

[fsn31324-bib-0055] Islam, M. T. (2017). Oxidative stress and mitochondrial dysfunction‐linked neurodegenerative disorders. Neurological Research, 39, 73–82. 10.1080/01616412.2016.1251711 27809706

[fsn31324-bib-0056] Johnson, E. J. , Mcdonald, K. , Caldarella, S. M. , Chung, H. , Troen, A. M. , & Snodderly, D. M. (2008). Cognitive findings of an exploratory trial of docosahexaenoic acid and lutein supplementation in older women. Nutritional Neuroscience, 11(2), 75–83. 10.1179/147683008x301450 18510807

[fsn31324-bib-0057] Jóźwiak, S. , Kossoff, E. H. , & Kotulska‐Jóźwiak, K. (2011). Dietary treatment of epilepsy: Rebirth of an ancient treatment. Neurologia I Neurochirurgia Polska, 45(4), 370–378. 10.1016/S0028-3843(14)60108-0 22101998

[fsn31324-bib-0058] Kalaiselvan, I. , Samuthirapandi, M. , Govindaraju, A. , Sheeja Malar, D. , & Kasi, P. D. (2016). Olive oil and its phenolic compounds (hydroxytyrosol and tyrosol) ameliorated TCDD‐induced heptotoxicity in rats via inhibition of oxidative stress and apoptosis. Pharmaceutical Biology, 54(2), 338–346. 10.3109/13880209.2015.1042980 25955957

[fsn31324-bib-0059] Karbowski, J. (2007). Global and regional brain metabolic scaling and its functional consequences. BMC Biology, 5, 10.1186/1741-7007-5-18 PMC1884139 17488526

[fsn31324-bib-0060] Kasarskis, E. J. , Berryman, S. , Vanderleest, J. G. , Schneider, A. R. , & McClain, C. J. (1996). Nutritional status of patients with amyotrophic lateral sclerosis: Relation to the proximity of death. American Journal of Clinical Nutrition, 63(1), 130–137. 10.1093/ajcn/63.1.130 8604660

[fsn31324-bib-0061] Koloverou, E. , Panagiotakos, D. B. , Pitsavos, C. , Chrysohoou, C. , Georgousopoulou, E. N. , Grekas, A. , … Stefanadis, C. (2016). Adherence to Mediterranean diet and 10‐year incidence (2002–2012) of diabetes: Correlations with inflammatory and oxidative stress biomarkers in the ATTICA cohort study. Diabetes/Metabolism Research and Reviews, 32(1), 73–81. 10.1002/dmrr.2672 26104243

[fsn31324-bib-0062] Kong, G. , Huang, Z. , Ji, W. , Wang, X. , Liu, J. , Wu, X. , … Zhu, Q. (2017). The Ketone Metabolite β‐hydroxybutyrate attenuates oxidative stress in spinal cord injury by suppression of class I histone deacetylases. Journal of Neurotrauma, 34(18), 2645–2655. 10.1089/neu.2017.5192 28683591

[fsn31324-bib-0063] Körner, S. , Böselt, S. , Thau, N. , Rath, K. J. , Dengler, R. , & Petri, S. (2013). Differential sirtuin expression patterns in amyotrophic lateral sclerosis (ALS) postmortem tissue: Neuroprotective or neurotoxic properties of sirtuins in ALS? Neurodegenerative Diseases, 11(3), 141–152. 10.1159/000338048 22796962

[fsn31324-bib-0064] Kouka, P. , Priftis, A. , Stagos, D. , Angelis, A. , Stathopoulos, P. , Xinos, N. , … Kouretas, D. (2017). Assessment of the antioxidant activity of an olive oil total polyphenolic fraction and hydroxytyrosol from a Greek Olea europea variety in endothelial cells and myoblasts. International Journal of Molecular Medicine, 40(3), 703–712. 10.3892/ijmm.2017.3078 28731131 PMC5547916

[fsn31324-bib-0065] Kruman, I. I. , Pedersen, W. A. , Springer, J. E. , & Mattson, M. P. (1999). ALS‐linked Cu/Zn‐SOD mutation increases vulnerability of motor neurons to excitotoxicity by a mechanism involving increased oxidative stress and perturbed calcium homeostasis. Experimental Neurology, 160(1), 28–39. 10.1006/exnr.1999.7190 10630188

[fsn31324-bib-0066] Lahoz, C. , Castillo, E. , Mostaza, J. , de Dios, O. , Salinero‐Fort, M. , González‐Alegre, T. , … Garcés, C. (2018). Relationship of the adherence to a mediterranean diet and its main components with CRP levels in the Spanish population. Nutrients, 10(3), 10.3390/nu10030379 PMC5872797 29558396

[fsn31324-bib-0067] Lukasova, M. , Malaval, C. , Gille, A. , Kero, J. , & Offermanns, S. (2011). Nicotinic acid inhibits progression of atherosclerosis in mice through its receptor GPR109A expressed by immune cells. Journal of Clinical Investigation, 121(3), 1163–1173. 10.1172/JCI41651 21317532 PMC3048854

[fsn31324-bib-0068] Mamelak, M. (2017). Energy and the Alzheimer brain. Neuroscience and Biobehavioral Reviews, 75, 297–313. 10.1016/j.neubiorev.2017.02.001 28193453

[fsn31324-bib-0069] Mancuso, R. , del Valle, J. Modol, L. , Martinez, A. , Granado‐Serrano, A. B. , Ramirez‐Núñez, O. , … Navarro, X. (2014). Resveratrol Improves Motoneuron Function and Extends Survival in SOD1G93A ALS Mice. Neurotherapeutics: the Journal of the American Society for Experimental NeuroTherapeutics, 11(2), 419–432. 10.1007/s13311-013-0253-y 24414863 PMC3996124

[fsn31324-bib-0070] Mattson, M. P. , Cutler, R. G. , & Camandola, S. (2007). Energy intake and amyotrophic lateral sclerosis. NeuroMolecular Medicine, 9, 17–20. 10.1385/NMM:9:1:17 17114821

[fsn31324-bib-0071] McGarry, J. D. , & Foster, D. W. (2003). Regulation of hepatic fatty acid oxidation and ketone body production. Annual Review of Biochemistry, 49(1), 395–420. 10.1146/annurev.bi.49.070180.002143 6157353

[fsn31324-bib-0072] Medina‐Remón, A. , Kirwan, R. , Lamuela‐Raventós, R. M. , & Estruch, R. (2018). Dietary patterns and the risk of obesity, type 2 diabetes mellitus, cardiovascular diseases, asthma, and neurodegenerative diseases. Critical Reviews in Food Science and Nutrition, 58, 262–296. 10.1080/10408398.2016.1158690 27127938

[fsn31324-bib-0073] Menzies, F. M. , Ince, P. G. , & Shaw, P. J. (2002). Mitochondrial involvement in amyotrophic lateral sclerosis. Neurochemistry International, 40(6), 543–551. 10.1016/S0197-0186(01)00125-5 11850111

[fsn31324-bib-0074] Miller, R. G. , Mitchell, J. D. , Lyon, M. , & Moore, D. H. (2003). Riluzole for amyotrophic lateral sclerosis (ALS)/motor neuron disease (MND). Amyotrophic Lateral Sclerosis and Other Motor Neuron Disorders, 4, 191–206. 10.1080/14660820310002601 13129806

[fsn31324-bib-0075] Morozova, N. , Weisskopf, M. G. , McCullough, M. L. , Munger, K. L. , Calle, E. E. , Thun, M. J. , & Ascherio, A. (2008). Diet and Amyotrophic Lateral Sclerosis. Epidemiology, 19(2), 324–337. 10.1097/EDE.0b013e3181632c5d 18300717

[fsn31324-bib-0076] Murata, T. , Ohtsuka, C. , & Terayama, Y. (2008). Increased mitochondrial oxidative damage in patients with sporadic amyotrophic lateral sclerosis. Journal of the Neurological Sciences, 267(1–2), 66–69. 10.1016/j.jns.2007.09.038 17961597

[fsn31324-bib-0077] Newman, J. C. , & Verdin, E. (2014). Ketone bodies as signaling metabolites. Trends in Endocrinology and Metabolism, 25, 42–52. 10.1016/j.tem.2013.09.002 24140022 PMC4176946

[fsn31324-bib-0078] Niessen, H. G. , Debska‐Vielhaber, G. , Sander, K. , Angenstein, F. , Ludolph, A. C. , Hilfert, L. , … Vielhaber, S. (2007). Metabolic progression markers of neurodegeneration in the transgenic G93A‐SOD1 mouse model of amyotrophic lateral sclerosis. European Journal of Neuroscience, 25(6), 1669–1677. 10.1111/j.1460-9568.2007.05415.x 17432958

[fsn31324-bib-0079] Offermanns, S. (2017). Hydroxy‐carboxylic acid receptor actions in metabolism. Trends in Endocrinology and Metabolism, 28, 227–236. 10.1016/j.tem.2016.11.007 28087125

[fsn31324-bib-0080] Okamoto, K. , Kihira, T. , Kondo, T. , Kobashi, G. , Washio, M. , Sasaki, S. , … Nagai, M. (2007a). Nutritional status and risk of amyotrophic lateral sclerosis in Japan. Amyotrophic Lateral Sclerosis: Official Publication of the World Federation of Neurology Research Group on Motor Neuron Diseases, 8(5), 300–304. 10.1080/17482960701472249 17852010

[fsn31324-bib-0081] Okamoto, K. , Kihira, T. , Kondo, T. , Kobashi, G. , Washio, M. , Sasaki, S. , … Nagai, M. (2007b). Nutritional status and risk of amyotrophic lateral sclerosis in Japan. Amyotrophic Lateral Sclerosis, 8(5), 300–304. 10.1080/17482960701472249 17852010

[fsn31324-bib-0082] Paganoni, S. , Deng, J. , Jaffa, M. , Cudkowicz, M. E. , & Wills, A. M. (2011). Body mass index, not dyslipidemia, is an independent predictor of survival in amyotrophic lateral sclerosis. Muscle and Nerve, 44(1), 20–24. 10.1002/mus.22114 21607987 PMC4441750

[fsn31324-bib-0083] Palamiuc, L. , Schlagowski, A. , Ngo, S. T. , Vernay, A. , Dirrig‐Grosch, S. , Henriques, A. , … René, F. (2015). A metabolic switch toward lipid use in glycolytic muscle is an early pathologic event in a mouse model of amyotrophic lateral sclerosis. EMBO Molecular Medicine, 7(5), 526–546. 10.15252/emmm.201404433 25820275 PMC4492815

[fsn31324-bib-0084] Paoli, A. , Bianco, A. , Damiani, E. , & Bosco, G. (2014). Ketogenic diet in neuromuscular and neurodegenerative diseases. BioMed Research International, 2014, 474296 10.1155/2014/474296 25101284 PMC4101992

[fsn31324-bib-0085] Paoli, A. , Cenci, L. , & Grimaldi, K. A. (2011). Effect of ketogenic Mediterranean diet with phytoextracts and low carbohydrates/high‐protein meals on weight, cardiovascular risk factors, body composition and diet compliance in Italian council employees. Nutrition Journal, 10(1), 112 10.1186/1475-2891-10-112 21992535 PMC3217855

[fsn31324-bib-0086] Paoli, A. , Moro, T. , Bosco, G. , Bianco, A. , Grimaldi, K. A. , Camporesi, E. , & Mangar, D. (2015). Effects of n‐3 polyunsaturated fatty acids (ω‐3) supplementation on some cardiovascular risk factors with a ketogenic mediterranean diet. Marine Drugs, 13(2), 996–1009. 10.3390/md13020996 25689563 PMC4344614

[fsn31324-bib-0087] Pehowich, D. J. , Gomes, A. V. , & Barnes, J. A. (2000). Fatty acid composition and possible health effects of coconut constituents. The West Indian Medical Journal, 49(2), 128–133. 10948851

[fsn31324-bib-0088] Pérez‐Guisado, J. , Mũoz‐Serrano, A. , & Alonso‐Moraga, Á. (2008). Spanish Ketogenic Mediterranean diet: A healthy cardiovascular diet for weight loss. Nutrition Journal, 7(1), 10.1186/1475-2891-7-30 PMC2586625 18950537

[fsn31324-bib-0089] Pérez‐Jiménez, J. , Elena Díaz‐Rubio, M. , & Saura‐Calixto, F. (2015). Contribution of macromolecular antioxidants to dietary antioxidant capacity: A study in the spanish mediterranean diet. Plant Foods for Human Nutrition, 70(4), 365–370. 10.1007/s11130-015-0513-6 26482738

[fsn31324-bib-0090] Perng, B. C. , Chen, M. , Perng, J. C. , & Jambazian, P. (2017). A Keto‐Mediet approach with coconut substitution and exercise may delay the onset of Alzheimer's disease among middle‐aged. The Journal of Prevention of Alzheimer's Disease, 4(1), 51–57. 10.14283/jpad.2016.104 29188860

[fsn31324-bib-0091] Polkey, M. I. , Lyall, R. A. , Moxham, J. , & Leigh, P. N. (1999). Respiratory aspects of neurological disease. Journal of Neurology Neurosurgery and Psychiatry, 66, 5–15. 10.1136/jnnp.66.1.5 PMC1736177 9886443

[fsn31324-bib-0092] Pradat, P.‐F. , & Dib, M. (2009). Biomarkers in amyotrophic lateral sclerosis. Molecular Diagnosis & Therapy, 13(2), 115–125. 10.1007/bf03256320 19537846

[fsn31324-bib-0093] Puchalska, P. , & Crawford, P. A. (2017). Multi‐dimensional roles of ketone bodies in fuel metabolism, signaling, and therapeutics. Cell Metabolism, 25, 262–284. 10.1016/j.cmet.2016.12.022 28178565 PMC5313038

[fsn31324-bib-0094] Rigacci, S. , & Stefani, M. (2016). May 31). Nutraceutical properties of olive oil polyphenols. An itinerary from cultured cells through animal models to humans. International Journal of Molecular Sciences, 17(6), 843 10.3390/ijms17060843 PMC4926377 27258251

[fsn31324-bib-0095] Robberecht, W. , & Philips, T. (2013). April). The changing scene of amyotrophic lateral sclerosis. Nature Reviews Neuroscience, 14, 248–264. 10.1038/nrn3430 23463272

[fsn31324-bib-0096] Robinson, A. M. , & Williamson, D. H. (2017). Physiological roles of ketone bodies as substrates and signals in mammalian tissues. Physiological Reviews, 60(1), 143–187. 10.1152/physrev.1980.60.1.143 6986618

[fsn31324-bib-0097] Rodríguez‐Morató, J. , Boronat, A. , Kotronoulas, A. , Pujadas, M. , Pastor, A. , Olesti, E. , … de la Torre, R. (2016). Metabolic disposition and biological significance of simple phenols of dietary origin: Hydroxytyrosol and tyrosol. Drug Metabolism Reviews, 48, 218–236. 10.1080/03602532.2016.1179754 27186796

[fsn31324-bib-0098] Rossi, M. , Caruso, F. , Kwok, L. , Lee, G. , Caruso, A. , Gionfra, F. , … Incerpi, S. (2017). Protection by extra virgin olive oil against oxidative stress in vitro and in vivo. Chemical and biological studies on the health benefits due to a major component of the Mediterranean diet. PLoS ONE, 12(12), e0189341 10.1371/journal.pone.0189341 29283995 PMC5746230

[fsn31324-bib-0099] Rossi, T. , Gallo, C. , Bassani, B. , Canali, S. , Albini, A. , & Bruno, A. (2014). Drink your prevention: Beverages with cancer preventive phytochemicals. Polskie Archiwum Medycyny Wewnetrznej, 124(12), 713–722. 10.20452/pamw.2560 25490889

[fsn31324-bib-0100] Ruskin, D. N. , Kawamura, M. , & Masino, S. A. (2009). Reduced pain and inflammation in juvenile and adult rats fed a ketogenic diet. PLoS ONE, 4(12), e8349 10.1371/journal.pone.0008349 20041135 PMC2796387

[fsn31324-bib-0101] Russo, M. A. , Sansone, L. , Polletta, L. , Runci, A. , Rashid, M. M. , De Santis, E. , … Tafani, M. (2014). Sirtuins and resveratrol‐derived compounds: A model for understanding the beneficial effects of the Mediterranean diet. Endocrine, Metabolic & Immune Disorders Drug Targets, 14(4), 300–308. 10.2174/1871530314666140709093305 25008762

[fsn31324-bib-0102] Samadi, M. , Moradi, S. , Moradinazar, M. , Mostafai, R. , & Pasdar, Y. (2019). Dietary pattern in relation to the risk of Alzheimer's disease: A systematic review. Neurological Sciences, 10.1007/s10072-019-03976-3 31240575

[fsn31324-bib-0103] Sarna, L. K. , Sid, V. , Wang, P. , Siow, Y. L. , House, J. D. , & O, K., (2016). Tyrosol attenuates high fat diet‐induced hepatic oxidative stress: Potential involvement of cystathionine β‐synthase and cystathionine γ‐lyase. Lipids, 51(5), 583–590. 10.1007/s11745-015-4084-y 26518313

[fsn31324-bib-0104] Shaw, P. J. , & Ince, P. G. (1997). Glutamate, excitotoxicity and amyotrophic lateral sclerosis. Journal of Neurology, 244(S2), S3–S14. 10.1007/bf03160574 9178165

[fsn31324-bib-0105] Silva, P. , Sureda, A. , Tur, J. A. , Andreoletti, P. , Cherkaoui‐Malki, M. , & Latruffe, N. (2019). How efficient is resveratrol as an antioxidant of the Mediterranean Diet, towards alterations during the aging process? Free Radical Research, 7, 1–12, 10.1080/10715762.2019.1614176 31039629

[fsn31324-bib-0106] Singh, R. , Sharad, S. , & Kapur, S. (2004). Free radicals and oxidative stress in neurodegenerative diseases: Relevance of dietary antioxidants. Journal of Indian Academy of Clinical Medicine, 5(3), 218–225.

[fsn31324-bib-0107] Song, W. , Song, Y. , Kincaid, B. , Bossy, B. , & Bossy‐Wetzel, E. (2013). Mutant SOD1G93A triggers mitochondrial fragmentation in spinal cord motor neurons: Neuroprotection by SIRT3 and PGC‐1α. Neurobiology of Disease, 51, 72–81. 10.1016/j.nbd.2012.07.004 22819776 PMC3992938

[fsn31324-bib-0108] Stafstrom, C. E. , & Rho, J. M. (2012). The Ketogenic Diet as a Treatment Paradigm for Diverse Neurological Disorders. Frontiers in Pharmacology, 3, 59 10.3389/fphar.2012.00059 22509165 PMC3321471

[fsn31324-bib-0109] Stanich, P. , Chiapetta, A. , Oliveria, A. , & Gabbai, A. (2002). Nutritional supplements in patients with amyotrophic lateral sclerosis. Amyotrophic Lateral Sclerosis Other Motor Neuron Disorders, 3(Suppl 2), 119.

[fsn31324-bib-0110] Steele, M. , Stuchbury, G. , & Münch, G. (2007). The molecular basis of the prevention of Alzheimer's disease through healthy nutrition. Experimental Gerontology, 42(1–2), 28–36. 10.1016/j.exger.2006.06.002 16839733

[fsn31324-bib-0111] Sun, Y. , Lu, C.‐J. , Chen, R.‐C. , Hou, W.‐H. , & Li, C.‐Y. (2015). Risk of amyotrophic lateral sclerosis in patients with diabetes: A Nationwide Population‐Based Cohort Study. Journal of Epidemiology, 25(6), 445–451. 10.2188/jea.je20140176 25947580 PMC4444499

[fsn31324-bib-0112] Taylor, M. K. , Swerdlow, R. H. , Burns, J. M. , & Sullivan, D. K. (2019). An experimental ketogenic diet for alzheimer disease was nutritionally dense and rich in vegetables and avocado. Current Developments Nutrition, 3(4), nzz003 10.1093/cdn/nzz003 PMC6435445 30931426

[fsn31324-bib-0113] Tefera, T. W. , Wong, Y. , Barkl‐Luke, M. E. , Ngo, S. T. , Thomas, N. K. , McDonald, T. S. , & Borges, K. (2016). Triheptanoin protects motor neurons and delays the onset of motor symptoms in a mouse model of amyotrophic lateral sclerosis. PLoS ONE, 11(8), e0161816 10.1371/journal.pone.0161816 27564703 PMC5001695

[fsn31324-bib-0114] Tieu, K. , Perier, C. , Caspersen, C. , Teismann, P. , Wu, D.‐C. , Yan, S.‐D. , … Przedborski, S. (2003). D‐β‐Hydroxybutyrate rescues mitochondrial respiration and mitigates features of Parkinson disease. Journal of Clinical Investigation, 112(6), 892–901. 10.1172/JCI200318797 12975474 PMC193668

[fsn31324-bib-0115] Tur, J. A. (2004). Los antioxidantes de la dieta mediterranea. Spanish Journal of Community, 4, 198–207. 10.1007/s00394

[fsn31324-bib-0116] Valko, K. , & Ciesla, L. (2019). Amyotrophic lateral sclerosis. Progress in Medicinal Chemistry, 58, 63–117. 10.1016/bs.pmch.2018.12.001 30879475

[fsn31324-bib-0117] Veech, R. L. (2014). Ketone ester effects on metabolism and transcription. Journal of Lipid Research, 55(10), 2004–2006. 10.1194/jlr.r046292 24714648 PMC4173993

[fsn31324-bib-0118] Veech, R. L. , Bradshaw, P. C. , Clarke, K. , Curtis, W. , Pawlosky, R. , & King, M. T. (2017). Ketone bodies mimic the life span extending properties of caloric restriction. IUBMB Life, 69, 305–314. 10.1002/iub.1627 28371201

[fsn31324-bib-0119] Vining, E. P. G. , Freeman, J. M. , Ballaban‐Gil, K. , Camfield, C. S. , Camfield, P. R. , Holmes, G. L. , … Wheless, J. W. (1998). A multicenter study of the efficacy of the ketogenic diet. Archives of Neurology, 55(11), 1433–1437. 10.1001/archneur.55.11.1433 9823827

[fsn31324-bib-0120] Wanders, D. , Graff, E. C. , White, B. D. , & Judd, R. L. (2013). Niacin increases adiponectin and decreases adipose tissue inflammation in high fat diet‐fed mice. PLoS ONE, 8(8), e71285 10.1371/journal.pone.0071285 23967184 PMC3742781

[fsn31324-bib-0121] Willett, W. C. , Sacks, F. , Trichopoulou, A. , Drescher, G. , Ferro‐Luzzi, A. , Helsing, E. , & Trichopoulos, D. (1995). Mediterranean diet pyramid: A cultural model for healthy eating. American Journal of Clinical Nutrition, 61(6), 1402S–1406S. 10.1093/ajcn/61.6.1402S 7754995

[fsn31324-bib-0122] Wong, P. C. , Pardo, C. A. , Borchelt, D. R. , Lee, M. K. , Copeland, N. G. , Jenkins, N. A. , … Price, D. L. (1995). An adverse property of a familial ALS‐linked SOD1 mutation causes motor neuron disease characterized by vacuolar degeneration of mitochondria. Neuron, 14(6), 1105–1116. 10.1016/0896-6273(95)90259-7 7605627

[fsn31324-bib-0123] Yang, S. Y. , He, X. Y. , & Schulz, H. (1987). Fatty acid oxidation in rat brain is limited by the low activity of 3‐ketoacyl‐coenzyme A thiolase. The Journal of Biological Chemistry, 262(27), 13027–13032. 3654601

[fsn31324-bib-0124] Youm, Y.‐H. , Nguyen, K. Y. , Grant, R. W. , Goldberg, E. L. , Bodogai, M. , Kim, D. , … Dixit, V. D. (2015). The ketone metabolite β‐hydroxybutyrate blocks NLRP3 inflammasome‐mediated inflammatory disease. Nature Medicine, 21(3), 263–269. 10.1038/nm.3804 PMC4352123 25686106

[fsn31324-bib-0125] Yudkoff, M. , Daikhin, Y. , Nissim, I. , Lazarow, A. , & Nissim, I. (2004). Ketogenic diet, brain glutamate metabolism and seizure control. Prostaglandins Leukotrienes and Essential Fatty Acids, 70(3), 277–285. 10.1016/j.plefa.2003.07.005 14769486

[fsn31324-bib-0126] Zhao, W. , Varghese, M. , Vempati, P. , Dzhun, A. , Cheng, A. , Wang, J. , … Pasinetti, G. M. (2012). Caprylic triglyceride as a novel therapeutic approach to effectively improve the performance and attenuate the symptoms due to the motor neuron loss in ALS disease. PLoS ONE, 7(11), e49191 10.1371/journal.pone.0049191 23145119 PMC3492315

[fsn31324-bib-0127] Zhao, Z. , Lange, D. J. , Voustianiouk, A. , MacGrogan, D. , Ho, L. , Suh, J. , … Pasinetti, G. M. (2006). A ketogenic diet as a potential novel therapeutic intervention in amyotrophic lateral sclerosis. BMC Neuroscience, 7, 29 10.1186/1471-2202-7-29 16584562 PMC1488864

[fsn31324-bib-0128] Zoccolella, S. , Simone, I. L. , Lamberti, P. , Samarelli, V. , Tortelli, R. , Serlenga, L. , & Logroscino, G. (2008). Elevated plasma homocysteine levels in patients with amyotrophic lateral sclerosis. Neurology, 70(3), 222–225. 10.1212/01.wnl.0000297193.53986.6f 18195267

